# A rare case rupture of sinus of Valsalva aneurysm in a peripheral hospital

**DOI:** 10.21542/gcsp.2024.41

**Published:** 2024-11-01

**Authors:** Naufal Fakhri Nugraha, Hilmi Nadya Hanifah, Zahra Nadiah, Nabila Azka Namirah, Abednego Panggabean

**Affiliations:** 1 Medical Doctor, Universitas Padjadjaran Hospital, Sumedang, West Java, Indonesia; 2 Medical Doctor, Hasna Medika Kuningan Heart Hospital, Kuningan, West Java, Indonesia; 3 Medical Doctor, Pameungpeuk Regional General Hospital, Garut, West Java, Indonesia; 4 Cardiologist, Pameungpeuk Regional General Hospital, Garut, West Java, Indonesia

## Abstract

**Background:** The sinus of Valsalva aneurysm (SVA) is an uncommon cardiac abnormality that may be either congenital or acquired.

**Case:** In this case we describe a 73-year-old man who complained of acute shortness of breath. Echocardiography showed rupturing of a sinus Valsalva aneurysm into the right ventricle with ventricular septal defect/rupture diagnoses in a peripheral hospital far from a reference hospital.

**Conclusion:** Echocardiography can aid in distinguishing between different diagnoses and serve as a catalyst for more exploration into the underlying cause. This can enhance the likelihood of early detection of an SVA and enable the implementation of an effective management strategy.

## Background

The sinus of Valsalva aneurysm (SVA) is an uncommon cardiac abnormality that may be either congenital or acquired^1^. Aneurysms occurring in the sinus of Valsalva exhibit a greater prevalence among males, with a male-to-female ratio of 4:1^2^. Echocardiography is the method of choice for the diagnosis of SVA. Unruptured sinus of Valsalva aneurysms (USVA) are usually found when identifying other cardiac lesions and patients are always asymptomatic. Acute rupture remains the most common and potentially most life-threatening complication^3^. Here, we present a rare case of a 73-year-old man rupturing a sinus of Valsalva aneurysm into the right ventricle with ventricular septal defect/rupture diagnoses.

## Case

A 73-year-old man complained of shortness of breath that had felt for three days before being admitted to the hospital. Complaints were accompanied by left chest pain that radiated to the back, feeling like a heavy weight was pressing on it and throbbing, which had felt since one week before entering the hospital. Complaints were also accompanied by fever and dizziness two days before entering the hospital. The patient admitted having a history of heart disease but did not know the exact diagnosis. The patient had not been under medical supervision for the past two years.

His initial vital signs showed a blood pressure of 110/65 mmHg, heart rate of 61 beats per minute, respiratory rate of 20 times per minute, temperature of 36.4 C, and saturation of 98% on room air. Physical examination was significant for a continuous cardiac murmur heard best on the right upper sternal border.

Laboratory data, including complete haematology, differential count of leucocytes, renal function, and glucose, were normal. There was an increase in the CK-MB enzyme of 47.0 U/L (normal value <24 U/L), and the HDL-lipid profile decreased by 39 mg/dL (normal value >60 mg/dL).

**Figure 1.**
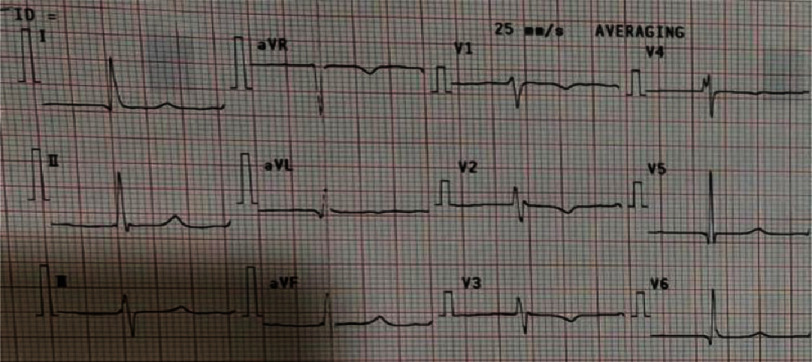
Electrocardiogram of patient on admittance.

**Figure 2.**
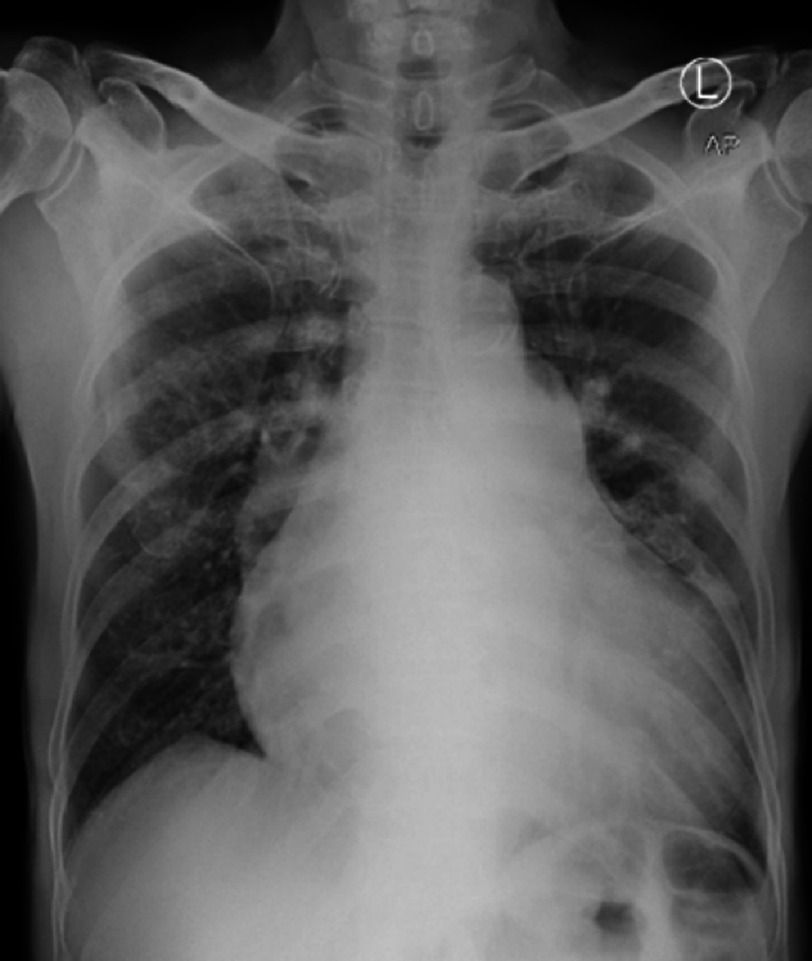
Postero-anterior chest X-ray.

An electrocardiogram (ECG) examination showed atrial fibrillation with normal ventricular response (AF NVR) (Figure 1). Postero-anterior chest X-ray showed an enlarged heart to the left side with the apex embedded in the diaphragm and the waist of the heart protruding, as well as signs of cranialization in pulmo, which gave the impression of cardiomegaly with signs of pulmonary oedema (Figure 2).

**Figure 3.**
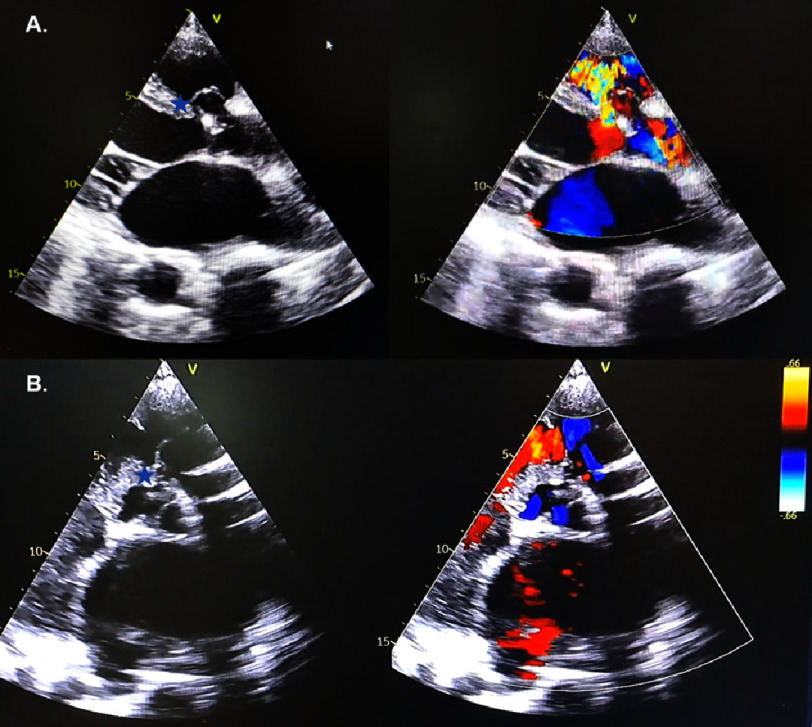
Echocardiography showing (A) PLAX view; (B) PSAX view. Blue star indicates interventricular septum gap with size 0.3 mm with shunt left to right).

Echocardiography examination showed dilated all chambers, reduced left ventricular systolic function with global hypokinetic, diastolic dysfunction, mild AR, intermediate probability of pulmonary hypertension, reduced RV contractility, ventricular septal defect (VSD) subaortic double commited ddx/ Ventricular Septal Rupture (VSR) 0.3 mm with sinus of Valsalva rupture drain to the right ventricle (Figure 3). The echocardiography video can be seen in Supplementary 1.

Considering the patient’s advanced age and life expectancy, the patient and family refused to be referred to a reference hospital for medical intervention and only chose to use symptomatic medication (Furosemide 2x40mg iv for early treatment, with Bisoprolol 1 × 1.25 mg, Spironolactone 1 × 25 mg, and Ramipril 1 × 2.5 mg after the patient was in a stable condition).

## Discussion

The diagnosis of sinus of Valsalva rupture can be challenging, particularly in peripheral settings. The patient’s symptoms when he first came to the hospital were secondary due to hemodynamic decompensation of the untreated aortic regurgitation related to SVA. Comprehensive physical examination and identification of a significant cardiac murmur, meant we recognized the urgent need for echocardiography to facilitate timely diagnosis. In peripheral settings, it is important for a cardiologist to give individual considerations in supporting echocardiography examinations as initial screening.

### Clinical characteristics

A rupture of a sinus of Valsalva is rare, mostly occuring in aneurysmal dilated sinuses. This cardiac lesion can be congenital or acquired. A congenital lack of continuity in the media, between the aorta and annulus fibrosus of the aortic valve, may initiate aneurysm formation or, less frequently, infection or degeneration processes may affect an aortic sinus. Unruptured sinus of Valsalva aneurysms are frequently asymptomatic and consequently seldom diagnosed. Sometimes prominent murmurs are generated, as blood flows in and out of an unruptured aneurysmal pouch. Cases of right ventricular outflow obstruction, coronary artery compression with infarction, conduction disturbances, endocarditis and thrombus within the aneurysmal cavity have also been reported^4^.

The sinus of Valsalva is a structure located within the aorta, specifically at the root of the ascending aorta. The sinus of Valsalva aneurysm (SVA) is characterized by the dilation or expansion of the aortic sinuses between the aortic valve annulus and the sinotubular junction^1^. The SVA has a notably low prevalence within the general population, with an incidence rate of 0.09%^5^ and with a male-to-female ratio of 4:1^2^. Most instances (ranging from 65% to 85%) have seen the probable emergence of aneurysms from the right coronary sinus. Subsequently, the noncoronary sinus has been identified as the origin in 10% to 30% of cases. Less commonly, aneurysms have been shown to form from the left coronary sinus, accounting for around 1% to 5% of cases^3^. One-third of cases resulting in rupture originate from the right coronary sinus. The presence of a rupture inside the chambers of the right heart can lead to a significant intra-cardiac shunt and the emergence of sudden heart failure^5^, often observed in individuals around the age of 40^6^.

The SVA can be categorized into two etiological types: acquired and congenital. Acquired aneurysms can arise from several causes, including trauma, tuberculosis, infective endocarditis, Behçet illness, syphilis, and atherosclerosis. Congenital aneurysms have been found to be linked with several cardiac abnormalities, including ventricular septal defects, bicuspid aortic valve, coronary artery anomalies, and aortic regurgitation^1^. It is worth contemplating the temporal correlation of concurrent illnesses, as they may not have arisen concurrently. The subpulmonic type of VSD, which is more prevalent in Asian populations and is associated with ruptured SVA, is the most typical type, followed by the perimembranous type, which is more typical in Western nations^3^. According to the available data, it has been shown that the subpulmonic variant of VSD has a higher prevalence among the Asian population compared to the Caucasian population, with a ratio of 5:1^7^. The SVA that ruptures into the right ventricle typically presents as a subpulmonic VSD or a perimembranous VSD, depending on how close to the pulmonic valve or tricuspid valve it enters^3^.

The SVA typically manifests with indications of aortic rupture, angina (caused by coronary artery compression), cardiac arrhythmias, or symptoms associated with the compression of adjacent tissues. According to recent research, atrial fibrillation was the most often reported arrhythmia, accounting for 9% of the cases examined. On the other hand, myocardial ischemia was identified in just five cases out of the total 53 recorded, and its occurrence was shown to be associated with larger dimensions of SVA^8^. The event of a ruptured SVA might manifest as an abrupt hemodynamic collapse. However, the presentation may vary in terms of its beginning, which can be more gradual, contingent upon the location and dimensions of the perforation^9^.

In this patient with unreported cardiac abnormalities, we hypothesize some mecahanism may contribute to the rupture of SVA: (1) The aorta has a lack of lamina elastic and muscular tissue, or an acquired weakening is observed in these regions^7^. The presence of a VSD is associated with the development of aneurysms. This is due to the same embryonic origin and congenital weakness of the ventricular septum and the right and non-coronary cusps in individuals with VSD. The condition known as SVA arises from the protrusion of the sinus of Valsalva into VSD, resulting in the creation of an aneurysmal abnormality^6^. There is a probability the occurrence of SVA is a chronic process due to the presence of AR; (2) The occurrence of a myocardial infarction in the past has resulted in a ventricular septal rupture, as a cardiac lesion^10^. This condition can lead to an elevated susceptibility to the development of infective endocarditis due to the presence of turbulent blood flow and possibility of colonization, hence contributing to the acquisition of SVA^1^; or (3) it could also be that the mechanism for the rupture of SVA and VSD/VSR occurs independently.

### Imaging diagnostic modalities

Various types of supporting examinations in diagnosing ruptured SVA have been used. Transthoracic echocardiography (TTE) is commonly employed as the primary diagnostic modality due to its non-invasive nature, cost-effectiveness, real-time capabilities, and ability to accurately assess dynamic anatomical structures, hemodynamics, and cardiac function^11^. TTE allows for a comprehensive evaluation of the aortic root, which may be visualized using parasternal long-axis, modified apical five-chamber, and three-chamber views. Additionally, the proximal ascending aorta can be assessed using the right parasternal long-axis, apical three-chamber, and suprasternal views^12^.

In SVA rupture, colour Doppler imaging reveals a persistent turbulent flow pattern between the aneurysm and the adjacent receiving chamber. Furthermore, TTE has revealed a characteristic “windsock” appearance when the burst section constitutes a significant proportion of the aneurysm. Notably, the observed “windsock” exhibits a dynamic behaviour, expanding and contracting in accordance with the cardiac cycle, as the flow through the ruptured section experiences fluctuations in its magnitude. On the other hand, a ventricular septal defect (VSD), which is frequently present concurrently, will have a systolic high-velocity jet and a diastolic low-velocity jet^13^.

Transesophageal echocardiography (TEE) can provide additional details on the architecture of the aortic root and the spatial relationship between the sinuses and adjacent structures^9^. However, it is often not recommended as the primary imaging modality for evaluating SVA because it is invasive. The proximity of the oesophagus to the aorta allows for acquiring high-resolution pictures of Valsalva’s aortic root and sinus using TEE imaging. These images are mainly obtained through the mid-esophageal aortic valve long-axis and mid-oesophagal aortic valve short-axis views. Transesophageal echocardiography (TEE) plays a crucial role in intervention procedures.

Multidetector cardiac computed tomography (MDCT) is a frequently employed imaging technique for diagnosing SVA and has been endorsed as the preferred imaging modality in specific recommendations, such as those provided by the Canadian Cardiovascular Society (CCS). The MDCT technique is an efficient diagnostic procedure that may obtain comprehensive data sets within a single breath hold. However, it is essential to consider that transportation to the scanner may not be feasible when current therapies and the patient’s condition are factors to be taken. Cardiovascular magnetic resonance (CMR) imaging has the potential to serve as a supplementary imaging technique in the identification of SVA and its associated consequences. The CMR has the advantage of evaluating cardiac function, such as the pulmonary-to-systemic blood flow ratio and aortic insufficiency^13^.

### Choices of treatment and challenges in peripheral areas

Surgical intervention is indicated for symptomatic, massive, or rapidly increasing unruptured SVA. Additionally, surgical management is recommended for unruptured SVAs that exhibit intraluminal thrombi, have a mass effect on adjacent tissues, or demonstrate recurrence. Surgical outcomes often show positive results, characterized by a favourable prognosis and modest recurrence rates^14^. The management of a ruptured SVA usually involves prompt cardiac surgical surgery. However, surgical treatment can increase other risks and even cause death. Additionally, patients will often optionally for an interventional approach if it can achieve the same results as surgery. Nevertheless, percutaneous approaches have also been employed and have had favourable results^15^.

Based on a recent systematic review and meta-analysis, no statistically significant differences were seen in terms of in-hospital mortality between percutaneous closure and surgical repair. However, it is noteworthy that percutaneous closure significantly reduced the average length of hospital stay compared to surgical repair^16^. Kuriakose et al. showed that PC might be safe, effective, and practical in patients who are too ill for bypass surgery and who have mild or no aortic regurgitation and simple associated defects (e.g., myxomatous ventricular septal defects, secondary foramen ovale septal defects, and small patent ductus arteriosus)^17^.

In the present study, our patient exhibited advanced age, with relatively stable condition and signs of heart failure. Our hospital does not have advanced modalities such as TEE, MDCT, or CMR to diagnose the patient. The nearest reference hospital we can reach was five to six hours away with difficult terrain and no air transport available. Advance age and socioeconomic status is also a consideration for the family to refuse further diagnostic testing and intervention in a reference hospital.

With advanced age, if the patient were able to undergo a procedure, percutaneous approaches would be preferred to reduce length of stay, faster recovery, and minimize hospital-acquired infection. According to the literature, the oldest age reported for a patient with SVA was 91 years, whereas the highest age recorded for a patient with surgery was 84 years^8^. The ruptured SVA exhibits a significantly unfavourable prognosis, with elevated morbidity and death rates^6^. Early surgical intervention is necessary for ruptured SVA due to a median untreated survival period of 3.9 years. Congestive heart failure is typically the primary cause of mortality^2^.

## What have we learned?

The occurrence of ruptured SVA is infrequent in the field of cardiology. It is essential for a cardiologist to give individual considerations in supporting echocardiography examinations as initial screening. Conducting an echocardiography in the early stages, mainly when the clinical presentation is ambiguous, can aid in distinguishing between different diagnoses and serve as a catalyst for more exploration into the underlying cause. This can enhance the likelihood of early detection of an SVA and enable the implementation of an effective management strategy.

## Supplementary video

Video: https://drive.google.com/drive/folders/1U9XeGNEZf8KDpdJEnSavSlfnDk4Z8xWq?usp=sharing

## Conflict of Interests

The authors declare that they have no conflicts of interest.

## Funding

This research has not received any specific grant from public, commercial, or non-profit sector agencies.

## Author Contribution

**Conceptualization:** Naufal Fakhri Nugraha, Hilmi Nadya Hanifah, Zahra Nadiah, Nabila Azka Namirah, and Abednego Panggabean

**Data Curation:** Naufal Fakhri Nugraha, Zahra Nadiah, and Nabila Azka Namirah

**Formal Analysis:** Naufal Fakhri Nugraha, and Hilmi Nadya Hanifah

**Investigation:** Naufal Fakhri Nugraha, Zahra Nadiah, Nabila Azka Namirah, and Abednego Panggabean

**Project Administration:** Naufal Fakhri Nugraha

**Supervision:** Abednego Panggabean

**Validation:** Naufal Fakhri Nugraha, Hilmi Nadya Hanifah, Zahra Nadiah, Nabila Azka Namirah, and Abednego Panggabean

**Writing –Original Draft:** Naufal Fakhri Nugraha, and Hilmi Nadya Hanifah

**Writing –Review and Editing:** Naufal Fakhri Nugraha, Hilmi Nadya Hanifah, Zahra Nadiah, Nabila Azka Namirah, and Abednego Panggabean

## References

[1] Jain A, Achuthan G (2019). Rupture of sinus of valsalva aneurysm into interventricular septum: Role of cardiac CT. Cureus.

[2] Doost A, Craig JA, Soh SY (2020). Acute rupture of a sinus of valsalva aneurysm into the right atrium: A case report and a narrative review. BMC Cardiovasc Disord.

[3] Togashi K, Paez FJG, Sheu RD (2020). Sinus of valsalva aneurysm rupture associated with a ventricular septal defect: The importance of multi-angle assessment by intraoperative transesophageal echocardiography. J Cardiothorac Vasc Anesth.

[4] Moreels S, Dymarkowski S, De Ridder S (2012). Ruptured sinus of valsalva in an asymptomatic patient - A case report. Eur Cardiol.

[5] Savill PJ, Rakhit DJ, Shah BN (2021). Ruptured sinus of valsalva aneurysm: Diagnosis by community echocardiography. Echo Res Pract.

[6] Morii Y, Morinaga H, Kato K, Hisagi M, Tanaka H (2023). A case of ruptured sinus of valsalva aneurysm in a patient with a ventricular septal defect who dropped out of lifelong medical follow-up. Internal Medicine.

[7] Arrascaeta-Llanes A, Kashyap A, Meyler D, Gupta R, Tharayil Z, Khan W (2020). Ruptured coronary sinus of valsalva in the setting of a supracristal ventricular septal defect. Clin Pract Cases Emerg Med.

[8] Marco LDi, Comentale G, Bruno M, Lanzillotti V, Colletta M, Russo V (2022). To treat or not to treat? This is the question... About the incidental finding of double sinus of valsalva aneurysm in a 91-year-old woman. Braz J Cardiovasc Surg.

[9] Kassab K, Kaul S, Gomez J, Delafontaine JL, Sawaqed R, Saini A (2021). Ruptured sinus of valsalva aneurysm: use of multimodality imaging in delineating structure and function. J Investig Med High Impact Case Rep.

[10] Zhang XY, Bian LZ, Tian NL (2021). The clinical outcomes of ventricular septal rupture secondary to acute myocardial infarction: A retrospective. Observational Trial. J Interv Cardiol.

[11] Jiang K, Chen J, Zhu X, Xiao H, Ran T, Tang Y (2022). Rupture of sinus of valsalva aneurysm: A case report in a child. BMC Cardiovasc Disord.

[12] Xu B, Kocyigit D, Betancor J, Tan C, Rodriguez ER, Schoenhagen P (2020). Sinus of valsalva aneurysms: A state-of-the-art imaging review. Journal of the American Society of Echocardiography.

[13] Arcario MJ, Lou S, Taylor P, Gregory SH (2021). Sinus of valsalva aneurysms: A review with perioperative considerations. Journal of Cardiothoracic and Vascular Anesthesia.

[14] Nguyen Q, Vervoort D, Phan K, Luc JGY (2021). Surgical management for unruptured sinus of valsalva aneurysms: A narrative review of the literature. J Thorac Dis.

[15] Duval JL, Ramsingh RAE, Rahaman NC, Rampersad RD, Angelini GD, Teodori G (2020). Rupture of sinus of valsalva aneurysm: Case report and review of contemporary literature. Perfusion.

[16] Mao Y, Wang C, Li Y, Guan X, Zhang X, Wu X (2023). Percutaneous closure versus surgical repair for ruptured sinus of valsalva aneurysm: A systematic review and meta-analysis. Frontiers in Cardiovascular Medicine.

[17] Kuriakose EM, Bhatla P, McElhinney DB (2015). Comparison of reported outcomes with percutaneous versus surgical closure of ruptured sinus of valsalva aneurysm. Am J Cardiol.

